# Minimally Invasive Radiofrequency Surgery in Sleep-Disordered Breathing

**DOI:** 10.3390/healthcare7030097

**Published:** 2019-08-18

**Authors:** Ankit Patel, Bhik Kotecha

**Affiliations:** ENT Department, Queen’s Hospital, Romford RM7 0AG, UK

**Keywords:** sleep apnoea syndromes, sleep-disordered breathing, minimally invasive surgery, radiofrequency surgery

## Abstract

Sleep-disordered breathing encompasses a spectrum of conditions ranging from simple snoring to obstructive sleep apnoea (OSA). Radiofrequency surgery represents a relatively new technique available to surgeons involved in managing this condition. Its principal advantage relates to its minimally invasive nature resulting in a reduced morbidity when compared to traditional sleep surgery. The presence of good-quality research evaluating the long-term outcomes is currently scarce, although the short-term data is promising. Careful patient selection appears to be paramount in obtaining a sustained improvement. The role of radiofrequency surgery in sleep-disordered breathing has been reviewed.

## 1. Introduction

Sleep-disordered breathing encompasses a spectrum of conditions ranging from simple snoring to obstructive sleep apnoea (OSA). The treatment of sleep-disordered breathing must be tailored to the individual. The initial management should include conservative measures, in the form of weight loss, reduction in alcohol, position therapy, and medical therapies such as topical nasal steroids [1]. More directed therapies such as mandible advancement devices and continuous positive airway pressure (CPAP) are useful in selected cases but limited by poor compliance. If these conservative measures fail, surgical intervention can be considered. The aim of surgery is to reduce airflow obstruction and turbulence during sleep. This obstruction can occur at multiple sites of the upper airway and, therefore, careful assessment is paramount to direct therapies appropriately [2]. Specifically, a drug-induced sleep nasendoscopy is performed to evaluate the nasal passages, nasopharynx, oropharynx, and hypopharynx during an induced sleep [3]. This allows the clinician to identify which sites to target—specifically aiming to shrink, stiffen, and stabilise the soft tissues [2]. As with all surgery, the aim is to undertake the least invasive procedure, with the fewest side-effects, fewest complications, and good long-term outcomes. There are many surgical options available to the sleep surgeon. The role of minimally invasive radiofrequency surgery has been reviewed. 

Radiofrequency ablation of the soft tissues was initially developed in the fields of cardiology, neurology, and urology [2]. A probe delivers high-frequency alternating current into tissue, causing ionic agitation. This subsequently causes local tissue heating, cell necrosis, and scarring. Histological studies have identified that the lesion spreads from the probe to an 8mm circumference [4]. This has the distinct advantage of providing therapeutic tissue necrosis in the interstitial layer but keeping the mucosal layer largely unaffected. The power transmitted to the tissues is regulated by an in-built algorithm within the generator. This ensures maximal tissue necrosis without charring [5]. In the initial phase, there is an acute inflammatory response manifested as pain and oedema; this is followed by collagen deposition and scarring at three weeks [2]. Further tissue volume reduction and scar contracture occur in the subsequent weeks, causing soft tissue stiffening and shrinkage. 

Radiofrequency can be used to fibrose tissue but also as a cutting technique with minimal collateral damage [6]. Radiofrequency acts without stimulating nerves and muscle, which allows it to be used without general anaesthetic in the clinic setting [4]. 

There are currently several manufacturers of radiofrequency generators used in sleep surgery—Celon^®^ (Olympus KeyMed Medical Industries and Equipment Ltd., Southend-on-Sea, UK), Coblator^®^ (Arthrocare Corp, Sunnyvale, CA, USA), Ellman^®^ (Oceanside, NY, USA), Somnus^®^ (Gyrus, Memphis, TN, USA),and Sutter^®^ (Fribourg, Germany). They vary in the frequency and polarity of high-frequency alternating current produced. Blumen et al. compared four of these generators to investigate whether the frequency or polarity had any effect on snoring outcome or pain. They concluded that all the generators were comparable in safety and efficacy [7]. Celon^®^ is our preferred generator because of its ability to provide both an interstitial probe and cutting probe [8]. 

## 2. Nose

Nasal obstruction is a contributing factor to sleep-disordered breathing in a number of patients [1]. This can be secondary to septal deviation, polyposis, or inferior turbinate hypertrophy. The role of radiofrequency is specifically to address inferior turbinate hypertrophy due to allergic or CPAP rhinitis, which has been refractory to medical treatment [2]. 

There are many alternative surgical methods to addressing the inferior turbinate including partial or total turbinectomy, linear cautery, submucous diathermy, or microdebrider-assisted turbinoplasty. However, these methods have been reported to cause increased pain, bleeding, and crusting [9].

Radiofrequency is applied to the submucous tissue, causing fibrosis of the underlying stroma, leaving the epithelium intact [9]. This reduces the inferior turbinate bulk in order to minimise nasal obstruction and improve sleep-disordered breathing in combination with other procedures. Typically, patients demonstrate an initial oedematous response for approximately one week and a subjective improvement in nasal obstruction after 2–3 weeks [10].

The principal advantages are that it can be performed relatively quickly, with minimal post-operative pain and the ability to perform under local anaesthetic in the clinic setting. Eighty-five percent of patients have some degree of crusting post-operatively, which settles within 4 weeks in the majority of cases [11]. Means et al. identified sustained improvement for >14 months following the procedure [11]. Atef et al. suggested that patients would have maximal reduction after more than one application [12], highlighting that second- or third-stage treatments are sometimes needed.

The comparison between techniques for reducing inferior turbinate hypertrophy has not been established with randomised controlled trials. Several observational studies have shown that microdebrider-assisted turbinoplasty is more effective at reducing nasal obstruction, but radiofrequency reduction produces fewer side effects [9]. 

### Suggested Technique

The procedure can be performed in the clinic under local anaesthesia or in combination with multilevel surgery under general anaesthesia. 

Lidocaine 2%+ 1:80,000 adrenaline is infiltrated into the submucous layer of the inferior turbinate. This assists in identifying the correct plane for probe insertion and reduces bleeding. 

Celon^®^ Pro-breathe probes are used at 15 watts. The needle is carefully inserted into the submucous layer of the inferior turbinate and passed posteriorly under direct vision. Care must be taken not to pierce the mucosa medially or exit the inferior turbinate posteriorly. Once the needle is in position, short applications of radiofrequency are made every 8mm as the needle is withdrawn. Under vision, the shrinkage of the inferior turbinate should be seen. Mucosal blanching or smoke in the nasal cavity indicates that the application is too superficial and the needle should be re-positioned into the submucosal plane. Incorrect application can result in increased crusting, adhesions, or mucosal erosions. In bulky inferior turbinates, two or more applications may be necessary—in these cases, the turbinate can be divided into a superior and inferior portion. Once complete, an antibacterial cream is applied to the turbinate anteriorly. 

## 3. Soft Palate

The soft palate contributes to sleep-disordered breathing in greater than 90% of cases [13]. There are several surgical procedures described for reducing the size of the soft palate, including uvulopalatopharyngoplasty, uvulopalatoplasty, and laser-assisted uvulopalatoplasty. These invasive procedures are associated with considerable post-operative morbidity [14]. The use of less-invasive radiofrequency treatment to the soft palate has been described since 1998 [15]. Since then, a multitude of studies have reviewed their use—although the radiofrequency device, number, and type of applications have varied [13,16]. The majority are observation studies showing promising but also variable results, which have been outlined below [16,17]. 

Radiofrequency treatment of the soft palate can be applied to interstitial tissue of the soft palate or used to resect excessive palatal tissue in the cutting mode [8,18].

The radiofrequency application causes a relatively minor reduction in volume of the soft palate. Its principal mechanism of action is thought to result from the increased scarring and subsequent stiffening of the soft palate, thereby reducing the collapsibility of the upper airway [17]. Back et al. found no statistical difference in the severity of mild obstructive sleep apnoea following a single radiofrequency application to the soft palate compared to placebo in a randomised controlled trial [17]. This highlights the need for careful patient assessment with drug-induced sleep nasendoscopy, the need to combine multilevel applications with other targeted sites, and the potential need for more than one application. On its own, this technique has been found to have a success rate at improving obstructive sleep apnoea of around 30% [19]. 

Atef et al. compared the use of radiofrequency versus laser-assisted uvulopalatoplasty for obstructive sleep apnoea. They concluded that both treatments required multiple treatments, but radiofrequency appeared to have a more sustained long-term outcome [20]. Back et al. reviewed the use of radiofrequency in snoring surgery and identified that the symptoms of snoring were reduced in the short-term, but long-term data was lacking [13]. Balsevicius et al. found that combined radiofrequency and uvulopalatoplasty was significantly more effective than radiofrequency alone in sleep-disordered breathing [21].

The principal advantage of this technique is related to the fewer side effects when compared to the more invasive alternatives. The major side-effects with radiofrequency use on the soft palate includes post-operative pain, superficial mucosal erosion, oedema, and palatal fistula. The incidence has been described at 3–4% [4,5]. Mucosal erosions have been attributed to poorly placed radiofrequency probes and generally heal spontaneously within two weeks [5]. Troell et al. identified that the mean post-operative pain duration was 2.6 days for radiofrequency compared to 13.8 days for laser-assisted uvulopalatoplasty [22]. Major adverse events or long-term side effects have not been reported [4]. It is widely accepted that a learning curve exists for this procedure, as complications appear to reduce with increasing experience [4,16]. No studies have reported velopharyngeal insufficiency, nasopharyngeal stenosis, or swallowing difficulty following the procedure [5]. 

Given the results of recent studies, we advocate the importance of careful patient selection prior to deciding which operative technique to employ on the soft palate. This surgery will almost always need to be performed in combination with other sites of the upper airway, which results in the best outcomes [4]. Similarly, it may require a second or third application. A patient with a particularly muscular soft palate may be better suited to having more invasive surgery (for example, laser-assisted uvulopalatoplasty) as radiofrequency treatment alone may have limited impact at stiffening the interstitial tissue. Likewise, to establish more successful results in selected cases, it is beneficial to reduce the uvula length by 50% and excise any redundant palatopharyngeus mucosa [8,18,21]. This acts to further improve the upper airway in a minimally invasive manner in combination with stiffening the soft palate. It also maintains the swallowing mechanism and reduces nasopharyngeal incompetence as compared to more invasive techniques. 

### Suggested Technique

This procedure is performed under general anaesthesia with an orotracheal or nasotracheal tube. A Boyle–Davis gag with Draffin rods is used to create an optimum surgical field. The mucosa is cleaned with chlorhexidine to reduce potential infection. Two tonsillar swabs are placed posterior to the soft palate to prevent inadvertent injury to the posterior pharyngeal wall [8,23]. 

Celon^®^ ProSleep Plus probes are used at 10 watts. Ten submucosal applications are ideally performed on the soft palate, although the exact number is dependent on the size of the palate. Lidocaine 2% + 1:80,000 adrenaline is infiltrated into the submucosal plane prior to application to reduce bleeding and increase the interstitial tissue volume to help identify the correct depth. Each application should be 8mm apart and carefully positioned in the interstitial palate tissue. Generally, two applications are placed 4mm from the midline, and three applications are placed 8mm lateral to these. The application should not cause mucosal blanching or exit the palate tissue posteriorly. 

Celon^®^ ProCut is used at 25 watts to reduce the uvula by 50% and excise the posterior tonsillar pillar mucosa. Care is taken to ensure the uvula is bevelled and that 2–3mm of mucosa is left between the posterior pillar and uvula [8]. Post-operation, five days of antibiotics are prescribed to prevent uvula infection.

## 4. Tonsil

Tonsillar tissue is a significant contributor to oropharyngeal obstruction and is present in the majority of sleep-disordered breathing patients. Generally, tonsillectomy using a variety of methods is the accepted technique in these cases, and results in effectively reducing obstructive symptoms. However, tonsillectomy is associated with significant post-operative pain, the risk of haemorrhage, and typically at least 1 or 2 weeks away from work. 

Tonsil reduction using radiofrequency treatment represents an alternative method. This technique causes soft tissue reduction whilst leaving the mucosa intact, similar to its effect on the soft palate and tongue base. By leaving the mucosa intact, the post-operative pain and risk of bleeding are significantly reduced. It can be performed under local anaesthetic or general anaesthetic in combination with multilevel surgery. Nelson et al. identified a rapid return to work within 1–2 days in a short-term study using the technique in nine patients [24]. In a further study, Nelson followed-up 12 patients for up to twelve months to demonstrate the long-term outcomes [25]. Statistically significant improvements in the oropharyngeal airway and sleep symptoms were identified. The results were sustained for 12 months. Six patients (50%) underwent a second procedure. Although all the patients had initial post-operative oedema, no patients had significant complications.

The use of radiofrequency to reduce tonsillar tissue appears to be safe and effective in selected patients. The conclusions are limited to small observational studies. Arguably, tonsillectomy is still preferable at excising large tonsils but limited by post-operative morbidity. The role of radiofrequency in tonsil reduction isnot completely clear but is potentially useful in patients who favour less invasive surgery, with smaller tonsillar tissue, as part of multilevel surgery. 

### Suggested Technique

This procedure is performed with an orotracheal or nasotracheal tube. The tonsils are exposed with a Boyle–Davis gag and Draffin rods similar to a conventional tonsillectomy. Celon^®^ ProSleep Plus is used at 10watts. The probe is inserted at three points into each tonsil from medial to lateral. The probe may need to be angled inferiorly depending on the size of the tonsil; this ensures that the radiofrequency is applied to the interstitial tonsillar tissue and not into the parapharyngeal space. 

## 5. Tongue Base

The tongue base contributes to approximately 25% of snoring at a single level and a larger proportion of snorers with multilevel narrowing [26,27]. The management of tongue base obstruction can be challenging because of difficult access and its functional importance in swallowing.

Non-surgical interventions include a mandibular advancement splint or chin strap. However, they are limited to patients with good dentition and are generally tolerated poorly [26]. Surgical interventions to address the tongue base or lower pharyngeal obstruction include mandibular osteotomies, partial tongue base resection, or hyoid suspension [28,29]. Although effective at treating obstructive sleep apnoea (OSA) in carefully selected patients, these procedures are particularly invasive and associated with significant morbidity. They are typically reserved for patients with severe OSA intolerable to CPAP as they are considered too extensive for less severe OSA and snoring.

Radiofrequency volume reduction of the tongue base was first described in 1999, and its effectiveness has been attributed to volume reduction but also tongue base stabilisation secondary to scarring [28,30]. 

Multiple observational studies have been undertaken to investigate the role of radiofrequency tongue base treatment in OSA and snoring. A recent systematic review has identified that in the short-term, the results are generally promising with improved objective and subjective features of obstructive sleep apnoea [28,30,31]. The longer-term results are limited but suggest that the initial improvement may reduce with time [28,31]. 

Initial side-effects in the form of tongue numbness, taste disturbance, and mild tongue weakness can occur, but generally settle within three weeks [4,28]. In 98 cases, Toh et al. had one case of tongue base haematoma which was managed conservatively [5]. In a total of 25 cases of tongue base radiofrequency, Pazos et al. had two cases of airway compromise due to tongue base oedema and two cases of tongue base abscess [18]. The use of chlorhexidine to clean the mucosal surface has been advised to reduce this risk. Tongue base ulceration, dysphagia, and crusting have been reported in case reports [5,18].

Welt et al. identified a poor sustained improvement in snoring following single modality radiofrequency treatment in patients with isolated tongue base hypertrophy [32]. This highlights the importance of dynamic airway assessment prior to any intervention. Patients who undergo this procedure should be carefully selected and identified to have tongue base narrowing. This procedure should also be combined with multilevel surgery as determined by the sleep nasendoscopy. Although the procedure can be performed under local or general anaesthesia, our preference is for general as this allows for a significantly improved visualisation and, thus, improved probe placement and improved efficacy [8,26].

This technique’s principal advantage relates to its minimal invasiveness, causing little pain, and the ability to perform under local anaesthetic in some institutions [32]. As with radiofrequency treatment to the soft palate, the disadvantages are associated with the recurrence in symptoms during long-term follow-up and the potential need for multiple treatments [26]. Arguably, in this regard, multiple applications in selected patients are acceptable given its low morbidity and can improve symptoms in a stepwise manner.

### Suggested Technique

The procedure is performed under general anaesthesia with a nasotracheal tube. The tongue base mucosa is cleaned with chlorhexidine to reduce the risk of post-operative infection. Obtaining an optimal view of the surgical field is essential to ensure effective placement of the radiofrequency electrode, which maximises efficacy and reduces complications. A Boyle–Davis gag and Draffin rods are used for optimal exposure. Exposure of the tongue base can be improved by pulling the tongue anteriorly prior to opening the Boyle–Davis gag. In addition, gentle external pressure on the mylohyoid can further optimise the view [8]. 

Celon^®^ ProSleep Plus is used at 6 watts. Six interstitial applications are typically applied to the tongue base, although the exact number is dependent on the size of the tongue base tissue and its involvement in the patient’s symptoms. This is carefully assessed during the pre-intervention sleep nasendoscopy. The probe should be directed perpendicular to the tongue surface and medially away from the neurovascular bundle. This reduces the risk of lingual nerve injury and haematoma. 

## 6. Complications

The specific complications and incidence relating to each site of radiofrequency application have already been discussed. Table 1 summarises the reported complications for each procedure. The relatively low incidence of complication is thought to result from the mechanism of radiofrequency. Radiofrequency causes ionic agitation and, therefore, heating of the surrounding tissues as opposed to the probe itself; this results in a predictable tissue injury pattern at a significantly lower temperature than direct electrocautery [23]. A histological study identified that the size of the lesion caused by interstitial radiofrequency is reliably an oval-shaped lesion, 6–7mm in width and 7–8mm in length [23]. The lesion size was independent of local anaestheticuse or power setting. No significant collateral tissue damage beyond this point was identified. Virk et al. subsequently advised that to avoid complications, radiofrequency applications should be made at least 8mm apart, and using local anaesthetic increases the interstitial volume, which reduces inadvertent mucosal erosion or fistulation [23]. Additionally, the radiofrequency probe must be fully inserted to prevent mucosal injury. The minimally invasive nature of the technique in addition to the minimal tissue damage results in the low complication rate. 

## 7. Conclusions

The presence of good-quality research which has investigated the long-term outcomes of radiofrequency in sleep-disordered breathing is currently scarce [34]. Veer et al. performed a systematic review to investigate the long-term outcomes of radiofrequency treatment in sleep-disordered breathing but found no randomised controlled trials [4]. From observational studies, Veer et al. identified that the large initial improvement of outcomes appears to reduce up to a year and then remain persistent. Baba et al. produced a systematic review summarising the efficacy of radiofrequency in obstructive sleep apnoea, and highlighted the importance of drug-induced sleep nasendoscopy for patient selection, where current data is limited by poor-quality methodologies, multilevel radiofrequency appears to have the most favourable outcome, and complications are infrequent [31].

The advantages of radiofrequency treatment for sleep-disordered breathing appear to be related to its minimally invasive nature, ability to perform under local anaesthetic, and low rate of complications. Patients may therefore find it acceptable to undergo second- and third-stage procedures to help create a sustained response. Many would argue that this is a more attractive option than undergoing more invasive upper airway surgery, although specific data to support these comments is lacking.

Minimally invasive radiofrequency surgery is one of many options in the armoury of the sleep surgeon to tackling upper airway obstruction. Careful assessment is paramount in selecting which patient will maximally benefit from this surgery. In the correct patient, radiofrequency surgery can provide sustained results without the unacceptable morbidity of more invasive procedures. Higher quality research is required to further validate its use and indications in sleep-disordered breathing.

## Figures and Tables

**Table 1:** Complications for radiofrequency surgery. * Not specifically reported, but possible from author’s experience.

Procedure	Possible Complications	References
Inferior turbinate	Bleeding, crusting * adhesions, infection	[9,11]
Soft Palate	Palatal oedema, infection, palatal erosion/ulcer, palatal fistula, globus * bleeding/haematoma, velopharyngeal insufficiency, dysphagia, pharyngeal stenosis, bleeding	[5,8,13,16,18]
Tonsil	* Bleeding	[24]
Tongue Base	Tongue base oedema (airway compromise), tongue ulceration, infection/abscess, haematoma, tongue weakness (hypoglossal nerve injury), taste alteration, tongue numbness, dysphagia	[5,8,18,23,33]
